# Older Patients’ Perspectives on Barriers and Facilitators to Counselling in Community Pharmacy: A Focus Group Study

**DOI:** 10.3390/healthcare14101354

**Published:** 2026-05-15

**Authors:** Rita Pedro, Rui Resende, Ana Reis, Ramona Mateos-Campos, Agostinho Cruz

**Affiliations:** 1 Faculty of Pharmacy, University of Salamanca, Campus Miguel de Unamuno, C. Lic. Méndez Nieto, s/n, 37007 Salamanca, Spain; rpa@ess.ipp.pt (A.R.); rmateos@usal.es (R.M.-C.); 2 REQUIMTE/LAQV, ESS, Polytechnic of Porto, Rua Dr. António Bernardino de Almeida, 4200-072 Porto, Portugal; agostinhocruz@ess.ipp.pt; 3 Escola Superior de Desporto e Lazer, Instituto Politécnico de Viana do Castelo, R. Dom Moisés Alves Pinho 4900, 4910-023 Viana do Castelo, Portugal; ruiresende@esdl.ipvc.pt; 4 SPRINT—Sport, Physical Activity and Health Research & Innovation Center—Centro de Investigação & Inovação do Desporto, 2040-413 Rio Maior, Portugal

**Keywords:** barriers, facilitators, community pharmacy, aged, focus group

## Abstract

**Highlights:**

**What are the main findings?**

- Regarding barriers, three main themes emerged: barriers centered in older adults, centered in physical space and centered in pharmacy professionals.
- Also, several facilitators were identified, which were organized according to the same three themes, with an additional one, facilitators centered in society.

**What is the implication of the main findings?**

- There is a significant gap regarding age-friendly environments and initiatives in community pharmacies settings.

**Abstract:**

**Background:** The ageing of world population is simultaneously a great triumph and a great challenge. It is essential to adjust healthcare systems to older populations and community pharmacies are not an exception. **Objectives:** The goal was to identify barriers and facilitators, perceived by older patients, that may influence counselling in community pharmacy and, consequently, therapeutic adherence. **Methods:** A qualitative study was performed. Six focus groups were conducted with 51 participants. The target population was people aged at least 65 years who are autonomous and frequently go to community pharmacies. **Results:** Three main themes emerged: barriers centered in physical space, centered in older adults and centered in pharmacy professionals. Eight categories emerged from the data analysis. Aisles and access, general impairments and time management were most mentioned categories of each theme, respectively. Furthermore, several facilitators were identified, which were organized according to the same three themes, with an additional one, facilitators centered in society. There were nine categories identified. Human skills, ambiance, appointment and medication management were the most cited ones of each theme, respectively. **Conclusions:** It is urgent to implement real measures to update community pharmacies settings. A checklist with the main facilitators and the barriers that should be avoided would help public authorities and community pharmacies directors to adapt and to change community pharmacies’ environments.

## 1. Introduction

The ageing of world population is simultaneously a great triumph and a great challenge [1]. Portugal is considered a superaged society [2]. In 2024, 24.1% of Portuguese population were 65 years or more, a higher number than the provisional percentage of European Union (EU) for the same year (21.6%) [3]. Despite being ambiguous, the definition of “older person” has evolved. In 2015, the World Health Organization (WHO) defined older person as “a person whose age has passed the median life expectancy at birth” and in 2025 as “a person aged 60 years or older” [4,5].

Portugal is one of an increasing number of countries worldwide to have committed to creating age-friendly environments [4,6,7]. In July 2025, Portugal approved the “*Estatuto da Pessoa Idosa* (Statute of the Elderly Person)” which is a set of measures that aims to assure active ageing with dignity and inclusion for older people. One of the main objectives is to reinforce autonomy and access to services with quality, with particular emphasis on mobility, accessibility and elimination of barriers [8,9]. In 2014, the Committee of Ministers of the Council of Europe published a recommendation about the promotion of human rights of older people. Among other recommendations, member states were advised to take appropriate preventive measures, to promote, maintain and improve the health and well-being of older people. In general, the recommendation outlines principles with a view to reinforcing the autonomy of older adults [10]. Also, back in 1991, United Nations general assembly adopted a resolution that defined the United Nations Principles for older people. Among others, principles like the ability to live in environments that are safe and adaptable to personal preferences and changing capacities or the integration in society, being an active part in the formulation and implementation of policies that directly affect their well-being are mentioned [11]. Thus, active ageing and the need to protect older people’s rights is not a recent theme.

In 2023, the average life expectancy at age 65 in the EU was estimated at 20.2 years, while Portugal outperformed this benchmark with an average of 21.1 years [12]. Even though life expectancy is improving, healthy life years have not had the same evolution: in Portugal, healthy years at 65 years old was 8.4 (in 2023), which means that people are living more but not necessarily living better [13,14,15]. “Adding years to life and life to years” is a challenging premise but behavior changes and strong innovations can actually occur when focus is combined with the right resources and with the collaboration of the main actors [16]. Thus, the concept of ageing in place has become more important as years go by. Ageing in place is the ability of older adults to stay safely and independently in their own houses and communities, whatever of age or ability [17]. WHO (2002) affirms that, among others, physical environment and personal determinants are fundamental to active ageing [1]. It is crucial to adapt healthcare systems to older population and community pharmacies are not an exception. Older adults have unique needs not only because of the necessity of multiple medications to delay and treat chronic diseases, but also because of age-related physiological changes. Pharmacy professionals are primary caregivers with privileged access to older adults [18]. The role of pharmacy professionals has become more important throughout the years, with professionals assuming increasingly complex roles and responsibilities [19,20]. Many professional organizations have already published guidelines on counselling referring to potential side effects, what to do with leftover medication and ensuring patients get all necessary therapeutic information, among other topics [21,22,23].

Communicational or architectural barriers can affect the relationship between pharmacy professionals and older patients and, consequently, the knowledge of the patient on medication, which can lead to adverse health outcomes. Thus, the identification and mitigation of barriers that limit this interaction is fundamental [19].

There are already some strategies that are implemented in some community pharmacies worldwide, but there is still a long way to achieve age-friendly cities and communities involving pharmaceutical environments [24,25]. Naturally, any changes implemented by these strategies will depend on the specific reality and legal framework of each country.

The World Health Organization and the United Nations declared 2021–2030 the Decade of Healthy Ageing. In this context, many reports, official documents and papers have emerged to try to help countries to discuss alternatives and to make changes to meet the ever-changing needs of our society [26]. The Green Paper on Ageing is one of these documents that discusses options on how to anticipate and respond to challenges and opportunities that ageing brings. Having a life-cycle approach, the document is based on the idea that all years since early childhood affect the rest of our lives. Thus, a healthy and active ageing should be promoted from the beginning, leading to a healthy lifestyle. The growing needs of the ageing population are also mentioned in this document, addressing policies such as urban renewal and accessibility in buildings. Although it is known (and reported) that older patients are more likely to suffer from chronic and multiple diseases, community pharmacies environments are not mentioned [27].

Portuguese authorities published an Action Plan for Active and Healthy Ageing that aims to guide and boost a transformation in Portuguese society. It is based in six strategic pillars, like “Health and Well-being” and “Autonomy and independent life” [28]. The first pillar focuses on the importance of promoting health and preventing disease. The second highlights accessible and age-friendly environments. The necessity of eliminating architectural barriers is mentioned but, once again, there is no mention of community pharmacies. It is urgent to support action on ageing at national, regional and local levels, to ensure that everyone can participate and be welcome in society.

This study involves older adults as primary participants, giving relevance to their perspectives and experiences, regarding attendance at community pharmacies. The goal was to identify barriers and facilitators perceived by older patients, that may influence counselling at a community pharmacy and, consequently, therapeutical adherence.

## 2. Materials and Methods

### 2.1. Study Design

This study employed an exploratory research approach utilizing focus groups. A focus group is a qualitative data collection technique that allows the investigator to achieve detailed data from a selected group of participants [29,30,31].

Qualitative research provides valuable insights into the reasons behind people’s actions or behaviors, allowing a deeper understanding of their underlying motivations [32].

The project was approved by the Ethics Committee of School of Health of Polytechnic of Porto (reference number CE0084B).

### 2.2. Participants

The target population was people of 65 or more years who are autonomous and frequently go to community pharmacies. Recruitment was conducted by direct contact with local authorities from the Oporto district that offer activities for seniors. After authorization, the research team went to classes to directly invite the elderly. The study was explained and, for those who were interested in, their names were registered. The session was scheduled based on participants’ preferences and local availability; the sessions occurred in the same place where older adults had gymnastic classes, to avoid mobility constraints. The sessions occurred in March 2024. In total, six focus groups were conducted, with a total of 51 participants.

Each participant’s number was assigned based on their focus group and participant number. For instance, participant 2.3 refers to the third participant in focus group two.

### 2.3. Focus Groups Design

Each session began with the reading and explanation of informed consent. All inquiries were taken, and all participants signed the informed consent. Each session lasted approximately 60 min; the audio was recorded to allow later transcription and data analysis. Focus groups discussions were conducted by a moderator. A script was prepared to develop and conduct the sessions in a structured way and was validated by an expert from Social and Human Sciences. It was designed based on short and clear questions, language that was understood by the target population and open-ended questions that promoted conversation and discussion about the topics [31].

The script consisted of six questions: an opening question, which was simple and direct to encourage participation (“Do you need to go to community pharmacies very often?”), a transition question (“Do you always go to the same community pharmacy? Can you explain why?”) and key questions, like “About physical space, do you detect any difficulty or obstacle in community pharmacies? How do you overcome these obstacles?” or “What characteristics do you consider a pharmacy professional should have to provide quality care to a patient with 65 years or more?”.

A pilot focus group was conducted to verify if the written script was correctly designed to generate the intended type of discussion. Since the goals were met, no modifications were deemed necessary.

### 2.4. Data Collection

The interviews were transcribed ad verbatim and the data were managed using NVivo (version 15), a qualitative data analytics software that allows the exploration and storage of high quantities of data [33]. The correlation between the data could be explored by using mental maps, coding matrix or word frequency [34]. In this study, the coding matrix was used to analyze the intervention of the participants in each theme and category and to verify if there were discrepancies in codification allowing deeper insight into the data. Also, mental maps were created to give a better general view of the identified themes and categories. Both mental maps and coding matrix are outputs of the NVivo software.

### 2.5. Analysis

The analysis was conducted following the thematic analysis proposed by Braun and Clarke (2012, 2014) [35]. After the transcription ad verbatim, initial categorization was performed, based on the main themes of the script. Subsequently, all focus group transcriptions were reviewed to distribute parts of the speech (units of reference) into categories. In the third phase of the process, all units of reference were revisited within each category to refine the process and assure adequacy [35,36]. The coding and categorization were completed by the main investigator with support and supervision from an experienced qualitative researcher. The categories were peer-reviewed by a critical friend who is defined as “*a trusted person who asks provocative questions, provides data to be examined through another lens, and offers critiques of a person’s work as a friend*” (Costa & Kallick, 1993, p. 2) [37]. A highly experienced pharmacy professional, with more than 15 years of professional experience, was invited. This process enabled the data to be examined from a different perspective, ensuring respectful and constructive feedback, and providing suggestions to clarify the results [38,39]. No study incentive was offered.

## 3. Results

A total of 51 older adults participated in the six focus groups discussions. Most participants were female (67%). This percentage aligns with expectations, as statistics indicate that women are more likely than men to use prescribed medicines; consequently, they are expected to visit community pharmacies more frequently.

From the analysis, three main themes emerged: (1) barriers centered in older adults, (2) barriers centered in physical space and (3) barriers centered in pharmacy professionals. In addition, several facilitators were identified, which were organized according to the same three themes, with an additional one, facilitators centered in society.

### 3.1. Barriers

**Figure 1:**
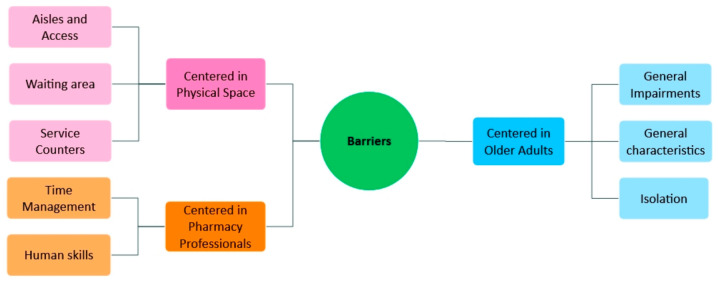
Mental map of barriers—Overview of the resulting themes and categories.

Regarding the identified barriers, three themes and eight categories emerged from the data analysis, which are represented in the mental map (Figure 1).

The coding matrix (Table 1) contains the themes and the total number of units of reference (represented in bold) and the categories with the corresponding number of units of reference for each group.

#### 3.1.1. Barriers Centered in Physical Space

Barriers centered in physical space were the most represented, comprising three categories and a total of 63 units of reference: (1) aisles and access; (2) waiting area; (3) service counters.

**Aisles and access.** This was the most mentioned category in this theme. It seems that the existence of exposed products leads to some discomfort in older patients because it influences the free space available in community pharmacy settings.

*It’s not that it makes me uncomfortable, but I don’t think it’s necessary to have the products exposed. We must see them, they are there to encourage a purchase. When you enter, you don’t have any free space, you must keep going around just to see the products.*

(Participant 2.11)

*Regarding physical space, there is a lot of products exposed… I find it confusing; there are marketing gondolas in the middle of the pharmacy with products, then another one further ahead with even more products, and so on until you reach the counter.*

(Participant 4.8)

**Waiting area**. The existence of chairs in the waiting area was highlighted as particularly important, because waiting times in pharmacies can be long.

*In the pharmacy where I go there are no chairs to sit in.*

(Participant 5.1)

*Some days ago, I was at the pharmacy, and there were 12 people waiting! Having chairs to wait is fundamental for older people.*

(Participant 7.5)

**Service counters.** This category had a strong connection with COVID-19 pandemic. The use of acrylic barriers at pharmacy reception desks was reported to affect communication.

*The acrylic barriers were always an obstacle to communication. The patient and the pharmacy professional were not at the same level. What happened was that people had to scream what they wanted. That really made me angry.*

(Participant 3.4)

*COVID was difficult for everyone, and it was hard at community pharmacy too. On one occasion, the doctor prescribed me new medication, and it was difficult for me to understand how to take it…*

(Participant 3.8)

#### 3.1.2. Barriers Centered in Older Adults

This was the second highest-mentioned theme and comprised three categories: (1) general impairments; (2) general characteristics; (3) isolation.

**General impairments.** This was the most prominent category within the theme of barriers centered in older adults. It included aspects such as hearing impairments, visual impairments, mobility issues and lack of perception. General impairments are more common and natural as time goes by. The existence of these impairments should be considered to not compromise the quality of counselling.

*If the patient had hearing difficulties, the pharmacy professional needs to speak louder.*

(Participant 2.3)

*Many people are too ashamed to admit it… For example, people who has visual impairments and leave their glasses at home…*

(Participant 2.8)

*Regarding mobility, it is also very different. A 30-year-old can quickly climb stairs, while a 70-year-old will go much slower.*

(Participant 5.1)

*Some people, when receiving information, must ask again, two or three times. This is a sign of a loss of perception.*

(Participant 4.7)

**General characteristics.** This category includes attributes of older population that can influence counselling like literacy, persistence or need for attention. The distinct kinds of characteristics of this category prove that older adults are a population that demands a specific type of treatment and counselling.

*There’s a huge difference between a 30-year-old and a 65-year-old, especially in communication. Everyone should be treated equally, even though they are different from each other. The pharmacy professional needs to know how to distinguish. If they realize they are dealing with someone with a certain level of education, they should use a different type of language.*

(Participant 3.4)

*Some people are shy, especially older people. Nowadays, young people have an education that we had not. Sometimes we use words that might not be appropriate for the occasion, but…*

(Participant 3.5)

*I think age makes things different. Young people should have more patience with us. I think we’re more picky, more persistent.*

(Participant 7.4)

**Isolation.** Isolation was identified by participants as an important feeling that can justify why older adults frequently go to community pharmacy to have some extra attention and to socialize.

*Perhaps the person who asks for the same information repeatedly also needs to have a little conversation with someone.*

(Participant 4.8)

*Some people go to the pharmacy just to talk with someone. But when this happens and there are a lot of patients waiting, I think pharmacy professionals should try to end the conversation quickly.*

(Participant 4.5)

#### 3.1.3. Barriers Centered in Pharmacy Professionals

This theme comprises two categories: (1) time management and (2) human skills.

**Time management.** Probably related to some general characteristics of older adults that were already mentioned, the waiting time was an important barrier mentioned by participants.

*When we go to the pharmacy, we can’t be in a hurry. Because waiting time can be too long!*

(Participant 2.8)

**Human skills.** During the interviews, some human skills of pharmacy professionals were mentioned as a barrier to counselling:

*“I realize that some pharmacy professionals don’t have a smile, it seems that there is no engagement…”*

(Participant 5.3)

*“I think that the oldest pharmacy professionals should have more empathy for the patient. Maybe it is because that already know the patient, but they are less friendly.”*

(Participant 7.3)

### 3.2. Facilitators

**Figure 2:**
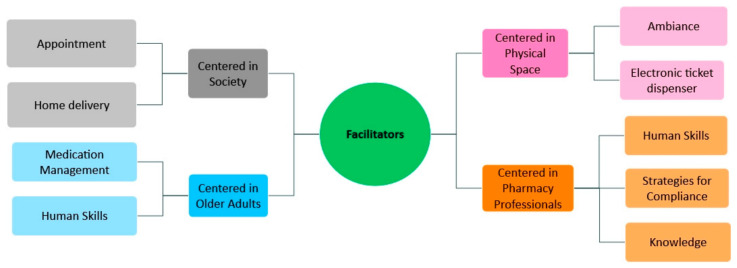
Mental map of facilitators—Overview of the resulting themes and categories.

During the data analysis, several facilitators were also identified and categorized using the same process. Four themes and nine categories were identified, which are represented in the mental map (Figure 2).

The coding matrix (Table 2) contains the themes and the total number of units of reference (represented in bold) and the categories with the corresponding number of units of reference for each group.

#### 3.2.1. Facilitators Centered in Pharmacy Professionals

Facilitators centered in pharmacy professionals were the most mentioned theme and comprised three categories: (1) human skills; (2) strategies for compliance; (3) knowledge.

**Human skills**. This category referred to the skills of pharmacy professionals and encompassed characteristics such as treating patients by name, active listening, empathy and sympathy.

*It also has a lot to do with the attendance at the community pharmacy. For example, with long-time patients being treated by name is very important.*

(Participant 2.8)

*There are many things that contribute to quality attendance at community pharmacies. I value being treated with eye contact and with a smile. If I don’t have that, I feel disappointed.*

(Participant 3.4)

*It must be empathic. Empathy is very important.*

(Participant 3.5)

**Strategies for compliance**. Several strategies to promote compliance were mentioned during the focus groups. These included practices such as repeating instructions and advice, asking patients for feedback to ensure that they understand every explanation and printing digital prescription.

*When the pharmacy professional realizes the patient hasn’t understood the information, they should use simpler alternative terms. The language should be adequate for the patient.*

(Participant 3.4)

*Especially if the patient can’t read, pharmacy professionals need to be sure that the patient has understood. They should ask “Did I make myself clear? Let’s recapitulate.”*

(Participant 3.4)

*Some modern community pharmacies print a label with posology. If the patient cannot read, the information must be given verbally.*

(Participant 3.7)

**Knowledge**. The ability of pharmacy professionals to counsel over-the-counter medications and to clarify medical advice was highlighted. Also, it became clear that many patients go to a community pharmacy in the first place in cases of minor health issues.

*Sometimes I go to community pharmacy without a prescription. When I present my symptoms, the pharmacy professional advises me, demonstrating knowledge and the advantages of some products over others. This demonstration of professional knowledge makes me feel secure!*

(Participant 2.8)

*I don’t like to go to the doctor. I go to community pharmacy and they have the knowledge, they say “take this, take that, …”*

(Participant 2.3)

#### 3.2.2. Facilitators Centered in Physical Space

Following the facilitators centered in pharmacy professionals, facilitators centered in physical space emerged as the most frequently mentioned theme. This theme comprises two categories: (1) ambiance; (2) electronic ticket dispenser.

**Ambiance**. Available space, luminosity, private room, chairs in the waiting area and parking facilities were some characteristics that older adults identify as an advantage.

*In the community pharmacy where I go, they have a private room to attend anyone who needs it.*

(Participant 5.6)

*For me, a good community pharmacy needs to have available space, marketing gondolas only in one side. It also needs to have chairs in the waiting area for people who might need it, like pregnant women, disabled people or people with canes.*

(Participant 5.1)

*Having parking facilities is also very important. Community pharmacies should have at least two or three private parking spaces.*

(Participant 7.3)

**Electronic ticket dispenser**. Electronic ticket dispensers were identified as a key facilitator, as they provide a structured queuing system that older patients find particularly helpful. Also, having a service counter specific to cosmetics was a suggestion to avoid higher waiting times in community pharmacy.

*At the community pharmacy where I go there’s space for everything. There’s an electronic ticket dispenser. We take a number and while we are waiting, we can see the products in the marketing gondola.*

(Participant 3.8)

*An attendance desk specifically for cosmetics would be ideal. If the patient needs a cosmetic, the pharmacy professional of this attendance desk will continue the counselling. Because waiting time in community pharmacy can be unpleasant.*

(Participant 5.3)

#### 3.2.3. Facilitators Centered in Society

This theme involves facilitators related to society and was divided into two categories: (1) appointment; (2) Home delivery.

**Appointment.** During the meetings, the idea of having a service by appointment came into conversation. Also, it was mentioned the need for frequent follow-up that could be done by pharmacy professionals, complementary to medical consultations with general doctors.

*The idea of scheduling the visit to a community pharmacy is interesting but not feasible in practice because everyone will be late!*

(Participant 4.7)

*Older patients with multiple pathologies stayed too long without a medical consultation. It should be a requirement for the patient to go to community pharmacies to measure blood pressure and other biochemical parameters. Values should be communicated to doctor’s system. This will allow a closer follow-up.*

(Participant 7.5)

**Home delivery**. Home delivery was a service considered important even though most of the participants affirm that they do not use it, which can be explained by the fact that being autonomous was one of the inclusion criteria of this study.

*Home delivery is an important service, even though I don’t use it!*

(Participant 2.7)

*If it was feasible for the community pharmacy, yes, it would be a good idea. It is a strategy for patient loyalty, and it gives them comfort. Obviously, it will be a disadvantage for those who like to go to community pharmacy to combat isolation.*

(Participant 4.7)

#### 3.2.4. Facilitators Centered in Older Adults

This was the least mentioned theme, also with two categories but with the lowest number of units of reference. It was possible to distinguish (1) medication management and (2) human skills.

**Medication management**. Community pharmacies that prepare the medication for patients are valued. Also, the advice and distinction between generic and brand medication was considered a facilitator.

*Sometimes I call for my community pharmacy, ask them to prepare for the medication and then I just pick it up. This service helps me a lot because I get medication for three different people.*

(Participant 2.5)

*Yesterday I went to community pharmacy, and they asked me if I wanted generic or brand-medication. The pharmacy professional advised me about the therapeutic effect, why one is more expensive than the other…*

(Participant 4.8)

**Human skills**. Patience, wisdom, being polite, maturity and experience were some of the older adults’ skills highlighted as facilitators to counselling.

*Between a 30-year-old patient and a 70-year-old patient everything is different. In every aspect and in every experience. Age is an encyclopedia, it brings wisdom and patience, it brings the ability to be polite and know how to wait. Nowadays, most young people are in a hurry, they want everything for yesterday. So, I think it is an advantage to be a 70-year-old patient.*

(Participant 5.4)

*I think age influences patience. Young people are more impatient. Older patients are more available.*

(Participant 6.4)

### 3.3. Thematic Overview

Table 3 shows an overview of the identified barriers and their corresponding facilitator.

## 4. Discussion

In this study it is clear the differences between the type of barriers mentioned. The most mentioned ones were barriers centered in physical space, englobing variables like aisles, access, waiting area and service counters. It is curious to note that most of the official documents and reports refer to the importance of eliminating architectural barriers and transforming cities into more age-friendly places. But these barriers are only related to outdoor spaces, like gardens or sidewalks [6,7]. Since the older population is more likely to have multiple diseases and, as a consequence, to experience polypharmacy, leaving community pharmacies out of barrier-free strategies is a major contradiction [18,40,41,42].

One of the highlighted barriers was the existence of marketing gondolas that take up most of the space in the community pharmacy. It will not be possible to eliminate the exposed products, because of the marketing campaigns, but having a limited number of exposed products according to the dimension of the pharmacy could be better for people with mobility impairments. The importance given to the existence of a proper waiting area should also be considered. Before the COVID-19 pandemic, the existence of chairs to sit in was standard practice. However, due to social distance measures, there was a reconfiguration of interior spaces, often resulting in the removal of waiting areas. In the post-pandemic context, the continued absence of these areas may negatively impact patient accessibility and comfort. Related to physical space, the acrylic barriers that some community pharmacies maintained even after the COVID-19 pandemic was also mentioned. Despite representing an additional measure to cleanliness for pharmacy professionals, it influences communication so its use should be avoided [43].

The second-most mentioned theme was barriers centered in older adults. Perhaps the advanced age gives self-conscience. It is not common to have participants in studies with such consciousness of how their characteristics can influence the process. It is important that pharmacy professionals have access to specific training on how to handle older patients’ characteristics to manage adequate attendance and counselling. General impairments like hearing, mobility or lack of perception are usual with the advance of age and should be considered. Some authors have already reported that hearing impairment can represent a risk to therapeutic adherence, due to the fact that medical instructions are not complete heard [44,45]. To overcome this barrier, pharmacy professionals should use strategies like repeating instructions or trying to obtain feedback to ensure that the patient understands all the given information [43,46].

In addition, older population may present some characteristics that should be considered when counselling, such as the level of literacy, persistence or need for attention. Established health literacy guidelines provide healthcare professionals with standardized strategies for effective patient communication. These frameworks offer the necessary tools to tailor counselling approaches based on the specific needs of individual patients, especially the older ones [47,48].

Isolation was also a self-perceived barrier that could explain the frequent community pharmacy visits of older patients. Social isolation and loneliness shorten older people’s lives and damage their physical and mental health. These consequences can be mitigated with specific strategies like face-to-face interventions or improved infrastructures that create age-friendly environments [49]. As one of the most accessible healthcare providers for the general population, pharmacy professionals play a crucial role in combating social isolation among older adults.

In this study there were also two barriers identified by older adults, applied to pharmacy professionals: time management and human skills. Time management could be a bit paradoxical, because older people, in general, are retired so it was expected that time management would not be a problem. Nevertheless, this perceived barrier could be explained by a sense of temporal urgency that is already characterized in the literature as an age-related behavioral frustration [50]. Having a smile, being empathic and being friendly are skills considered indispensable to provide high quality patient care. It is fundamental that pharmacy professionals have specific training on how to interact with patients, especially the most vulnerable ones [47].

The importance of identifying barriers is unquestionable but finding ways to mitigate them is a crucial question. In this study it was possible to relate some facilitators to the identified barriers. To face some barriers related to physical space like absence of a waiting area or aisles and access full of exposed products, the participants mentioned the importance of having enough space, with good luminosity and chairs in the waiting area (category ambiance, centered in physical space).

The participants identified many characteristics of themselves that are considered barriers to counselling, like education level, language or need for attention. To overcome these barriers, some facilitators centered in pharmacy professionals were mentioned, like strategies for compliance, human skills and knowledge. In addition, general impairments and isolation were identified barriers centered in older adults that could be overcome by the same facilitators centered in pharmacy professionals.

Time management was an identified barrier centered in pharmacy professionals that could be overcome by a curious facilitator that was identified as centered in society: the possibility of making an appointment in the pharmacy. Also, older patients identified existing professional skills among pharmacy staff that could represent a barrier and suggested additional interpersonal qualities that could help overcome the identified barriers.

Not all barriers had corresponding facilitators, and vice versa. Consequently, some facilitators were discussed during the focus groups even when their associated barriers were not identified, which reinforces the need for future studies. Most facilitators were mentioned when the main investigator asked, “*If you had the power to decide, what would you do differently at your regular pharmacy?*”.

There are already initiatives in place to make the community pharmacy environment more age friendly. The John A. Hartford Foundation and the Institute for Healthcare Improvement (IHI), in partnership with the American Hospital Association (AHA) and the Catholic Health Association of the United States (CHA), designed the Age-Friendly Health System, which follows an essential set of evidence-based elements, known as the “4Ms”: what matters, medication, mentation and mobility. “What matters” is about the importance of understand health goals and preferences of older adults and to find ways to optimize what matters; “medication” is related to conversations with older adults and their family caregivers about medication; and “mentation” focuses on monitoring mental and cognitive well-being of older adults. Finally, “mobility” is about move safely and improve function. It is a movement to help hospitals, medical practices and others to deliver age-friendly care. Portugal already has three recognized healthcare sites [51,52,53].

The present qualitative study clearly highlights three of the 4Ms: “what matters”, since the study not only allowed the identification of barriers but also the identification of facilitators; “mentation”, because isolation was identified as a barrier centered in older adults; and “mobility”, due to the fact that lack of mobility was mentioned as a general impairment and also older adults mentioned some discomfort related to the exposed products in the aisles and accesses. Implementing the 4Ms in community pharmacies should be a priority.

Also, Malet-Larrea and colleagues (2019) [25] developed a study that aimed to define and implement the concept of an “Age-friendly Pharmacy”, which had fifteen criteria clustered into four topics: relationships, pharmacy layout, pharmaceutical services and communication of services. It is curious to note that most of the criteria that are included in that study, identified as essential for an age-friendly pharmacy, are mentioned in the present study, including pleasant treatment, personalized attention and having an attentive pharmacy professional who gives attention based on trust and respect. Related to pharmacy layout, the importance of having no architectural barriers, ensuring privacy and having chairs in the waiting area were also common points of both studies, among others [25].

In recent years, the professional role of pharmacy professionals has had a significant change, particularly within the domain of geriatric care. Historically, the pharmacy professionals’ role was largely circumscribed to medication dispensing and over-the-counter (OTC) counselling. However, as some of the most accessible practitioners within the healthcare system, pharmacy professionals now fulfill a crucial role by having a measurable impact through community-based interventions designed to optimize pharmacotherapy, enhance medication management and address polypharmacy through proactive recommendations [54,55,56].

Having a study like this, whose participants are the older population itself, and having these many barriers highlighted should be enough to at least make authorities consider the urgence of creating a checklist or a good practice manual for empowering community pharmacy managers to reorganize physical spaces and operational structures, addressing identified barriers and implementing respective facilitators.

### Strengths and Limitations

The strengths justify the relevance of this study. First, because it prioritizes the patients’ perspectives, particularly regarding vulnerable cohorts like the older population. Usually, investigations focus on health professionals. Furthermore, this study had a significant number of participants, which enhances the findings.

One limitation of this study is the inherent difficulty in generalizing the qualitative data that may lead to an eventual subjective interpretation. This limitation was overcome by the involvement of a critical friend to ensure rigor. Being an exploratory study, the aim was to develop interpretations that lead to deep insights and studies, rather than generalize findings to similar populations [57]. Future research can overcome this limitation by utilizing larger cohorts to validate these thematic insights.

## 5. Conclusions

Population ageing is one of humanity’s greatest triumphs. It is also one of our greatest challenges. Much work is already done regarding age-friendly environments, but there is a significant gap regarding community pharmacies’ settings. The world’s response to COVID-19 illustrates how rapid innovation and widespread behavior change can occur when concentrated attention is supported by sufficient resources and collaboration. Thus, the same strategy could be used for age-friendly environments, with a particular focus on the community pharmacy setting. It is urgent to implement real measures to update community pharmacies settings. A checklist with the main facilitators and the barriers that should be avoided would help public authorities and community pharmacies directors adapt and change community pharmacy environments.

## Figures and Tables

**Table 1:** Coding matrix of barriers: Overview of the resulting themes and categories that emerged from focus groups.

	FG2	FG3	FG4	FG5	FG6	FG7	Total
Centered in Physical Space	**11**	**22**	**8**	**11**	**7**	**4**	**63**
Aisles and Access	8	7	5	3	5	2	30
Waiting Area	3	9	3	6	2	2	25
Service Counters	0	6	0	2	0	0	8
Centered in Older Adults	**3**	**2**	**9**	**2**	**6**	**6**	**28**
General Impairments	3	0	5	2	3	2	15
General Characteristics	0	2	0	0	2	3	7
Isolation	0	0	4	0	1	1	6
Centered in Pharmacy Professionals	**4**	**2**	**6**	**2**	**2**	**1**	**17**
Time Management	4	2	6	1	2	0	15
Human Skills	0	0	0	1	0	1	2

(FG: Focus group).

**Table 2:** Coding matrix of facilitators: overview of the resulting themes and categories that emerged from focus groups, with the total number of units of reference for each group.

	FG2	FG3	FG4	FG5	FG6	FG7	Total
Centered in Pharmacy Professionals	**17**	**18**	**9**	**19**	**9**	**13**	**85**
Human skills	5	11	7	10	8	8	49
Strategies for compliance	5	6	2	4	1	3	21
Knowledge	7	1	0	5	0	2	15
Centered in Physical Space	**4**	**1**	**2**	**8**	**3**	**11**	**29**
Ambiance	3	0	1	3	2	11	20
Electronic ticket dispenser	1	1	1	5	1	0	9
Centered in Society	**6**	**0**	**8**	**1**	**0**	**3**	**18**
Appointment	2	0	4	1	0	3	10
Home delivery	4	0	4	0	0	0	8
Centered in Older Adults	**3**	**2**	**2**	**4**	**3**	**0**	**14**
Medication management	3	1	2	2	0	0	8
Human skills	0	1	0	2	3	0	6

(FG: Focus group).

**Table 3:** Overview of the identified barriers and facilitators that may overcome these barriers.

Barriers	Facilitators that May Overcome the Identified Barriers
Centered in Physical Space	
Aisles and Access	Ambiance (Centered in Physical Space)
Waiting Area	Ambiance (Centered in Physical Space)
Service Counters	Non defined
Centered in Older Adults	
General Impairments	Strategies for compliance (Centered in Pharmacy Professionals)
General Characteristics	Human skills and Knowledge (Centered in Pharmacy Professionals)
Isolation	Human skills and Knowledge (Centered in Pharmacy Professionals)
Centered in Pharmacy Professionals	
Time Management	Appointment (Centered in Society)
Human Skills	Human skills (Centered in Pharmacy Professionals)

## Data Availability

The raw data supporting the conclusions of this article will be made available by the authors on request.

## Abbreviations

The following abbreviations are used in this manuscript:

AHA	American Hospital Association
CHA	Catholic Health Association of the United States
COVID-19	Coronavirus disease 2019
EU	European Union
FG	focus group
IHI	Institute for Healthcare Improvement
OTC	over-the-counter
WHO	World Health Organization
